# Neural Information Processing in Cognition: We Start to Understand the Orchestra, but Where is the Conductor?

**DOI:** 10.3389/fncom.2016.00003

**Published:** 2016-01-26

**Authors:** Günther Palm

**Affiliations:** Institute of Neural Information Processing, University of UlmUlm, Germany

**Keywords:** computational neuroscience, artificial intelligence, large scale computational modeling, computational cognition, cognitive neurocomputing

## Abstract

Research in neural information processing has been successful in the past, providing useful approaches both to practical problems in computer science and to computational models in neuroscience. Recent developments in the area of cognitive neuroscience present new challenges for a computational or theoretical understanding asking for neural information processing models that fulfill criteria or constraints from cognitive psychology, neuroscience and computational efficiency. The most important of these criteria for the evaluation of present and future contributions to this new emerging field are listed at the end of this article.

## The Development of the Field of Neural Information Processing

Beginning with the theoretical foundations of cybernetics and information theory by Wiener (1948) and Shannon (1948), the field of theoretical neuroscience started to develop in the direction of neural information processing. At that time, scientists were inspired by the idea that the same theoretical ideas can be employed both in technological developments and in the understanding of biological, environmental or even sociological systems. Many new concepts in the areas of control, pattern recognition, sensory and motor physiology, neurology, and brain research in general were invented, and it often remains obscure whether the first inspiration comes from biological or neurological observations on one hand or from cybernetical or engineering inventions on the other. An example is the idea of the “receptive field” of neurons in the visual system and of edge or line detectors, called “simple cells”, in particular, that were substantiated by the Nobel prize winning research of the neurophysiologists Hubel and Wiesel (e.g., Hubel and Wiesel, 1968; Hubel et al., 1977).

After the initial phase of concerted progress towards both technical applications and neuroscientific insights, the field of neural information processing started to split into technology oriented research (neurocomputing and computational learning theory) and neuroscience oriented research (computational neuroscience) in the 1980s.

On the technological side the driving forces or “biological inspirations” came from two observations:

1. The brain consists of a huge number of computational units (the neurons) working in parallel, apparently without a substantial amount of coordinating or synchronizing “overhead”. This inspired computer scientists to think in the direction of non-conventional massively parallel computing architectures (e.g., Palm and Palm, 1991; Heittmann and Rückert, 1999; Zhu and Hammerstrom, 2002). Unfortunately, many of these early ideas were not so successful in terms of applications, because during the time needed to develop such architectures, algorithms, and programming languages for them, the available conventional computing hardware became 100–1000 times faster (“Moore’s law”), easily equaling or surpassing the speed-gain achieved by new massively parallel architectures. This trend, however, seems to come to an end these years, and consequently during the last 5–10 years many of the “old” ideas concerning massively parallel architectures (e.g., in Krikelis and Weems, 1997 or in Ramacher et al., 1991) have been rediscovered or reinvented. Notably, this development of new unconventional computational architectures is declared as one major goal of the new large European Human Brain Project (HBP), the other, of course, being the understanding of the human brain.
2. The neurons in the brain are able to learn (mostly by synaptic plasticity, i.e., by changing the weights of the network connections). The first successful applications of artificial neural networks were based on this learning ability. The neural network architectures used in these days were utterly simple (mostly 2- or 3-layer feedforward networks with supervised learning by gradient descent) and the rule for changing the connections was not so close to biological reality, but the learning was often successful leading to useful applications and it still was in many ways similar to or “inspired by” learning processes in real neurophysiological networks.

During the 90’s and to a large degree initiated and guided by the mathematically beautiful statistical learning theory developed by Vapnik (1998), the practical and technical approaches to learning systems became much more sophisticated (for an overview, see Bishop, 2006), but the new machinery used for learning lost much of its direct appeal to neuroscience. On the other hand, the architectures employed for learning have become more complex and modular (e.g., “deep” multilayer networks, Bengio, 2009; Bengio et al., 2013; Abdel-Rahman et al., 2012; Hinton et al., 2012; LeCun et al., 2015), involving hierarchical configurations and even recurrent networks (reservoir computing, e.g., Maass et al., 2002; Jaeger and Haas, 2004), and also the forms of learning have become modular and perhaps more “biological”, moving from “supervised” learning (requiring a “teacher signal” or “label” for the data points) towards various combinations of supervised, partially supervised, and unsupervised learning (e.g., Chapelle et al., 2006; Schwenker and Trentin, 2014). Many of these more complex architectures and mechanisms for learning now require a scheduling of the order or sequence in which (different parts of) the learning material is presented to different parts of the network, reminiscent of the “critical” periods (of presumably higher synaptic plasticity) known in neuroscience, whose timing may differ in different areas of the cortex.

On the neuroscientific side, neural modeling has been vastly extended, both in detail and in size. In particular, the processes and mechanisms involved in the spatio-temporal integration of activity in the dendrites (e.g., backpropagation of the spike; Stuart and Sakmann, 1994; Markram et al., 1995; Stuart et al., 1997) and in synaptic transmission, encompassing various forms of synaptic plasticity at several time-scales (e.g., STDP, Markram et al., 1997; Bi and Poo, 1998; Ziegler et al., 2015), have been investigated, analyzed, and simulated in more detail. Notably these simulations have often added a “computational” or functional dimension concerning the potential use or purpose of such mechanisms to a purely descriptive biophysical analysis. On the other hand, much larger networks of more simplified model neurons could be simulated due to the quickly increasing available computer power. Such larger scale or even “systemic-level” simulations were mostly guided by functional or computational ideas concerning information processing in neural networks that is able to realize interesting behavioral or even cognitive functionalities.

These investigations have led to the establishment of the discipline of computational neuroscience as evidenced by several books, conferences, and journals (e.g., Churchland and Sejnowski, 1992; Dayan and Abbott, 2001; Arbib, 2002; the NIPS conferences, Journals “Neural Computation”, “Neurocomputing”, “Neural Networks”).

An important issue within the community of computational neuroscience has been “neural coding” and more generally the use of information theory in the evaluation of single neuron responses and neural networks. The question of the neural code has been debated heatedly since the late 1960s (Perkel and Bullock, 1967). The main issue was the interpretation and use of neural spike responses in terms of single spike timing or spike frequency evaluation. Much of this research was driven by the perhaps naive question, why the brain uses spikes for communication and maybe also for computation. Many sensor and motor functions have been implemented by networks of spiking neurons and there are large-scale hardware realizations for this (e.g., Mead, 1989; Mahowald and Douglas, 1991), some of which are currently under development (e.g., Merolla et al., 2014 or in the HBP^1^, Markram et al., 2015), with the vague prospect of being useful for technical applications. For hardware realizations of associative memories spiking activity may be useful because it fits well with the required sparseness of activity patterns (Palm, 2013). Also correlations or synchrony of activity may be easier to compute by counting coincident spikes (see the literature on “binding”, e.g., Engel and Singer, 2001). However, no convincing general theoretical argument for a principled computational advantage of spikes vs. continuous potentials has been put forward yet.

In this context and also in the analysis and evaluation of peripheral (sensory or motor) neural responses and of the storage and retrieval capacity of rules for synaptic plasticity, information theory, in particular maximization of mutual information, was often used in computational neuroscience, neural modeling and sometimes even in experimental neuroscience (see Grün and Rotter, 2010; and citations in Palm, 2012, ch. 12).

On the technical side this is paralleled by a quite common use of ideas from information theory in neurocomputing and learning theory, in particular the use and optimization of the logarithm of *a posteriori* probabilities or the Kullback-Leibler information distance for the derivation of learning rules (citations in Palm, 2012, ch.11) and the interpretation of neural activation as Bayesian inference (e.g., Rao et al., 2002; Doya et al., 2006).

## New Challenges

The further we move away from the periphery into central information processing and true human cognitive abilities, the sparser gets the amount of insight or inspiration we can find in current computational neuroscience. At the same time many sophisticated behaviors now are labelled as “cognitive”, which is often far from the original meaning (cf. Webster’s). There are some computational ideas concerning mirror neurons and language processing (e.g., Yu and Ballard, 2004; Arbib, 2006; Markert et al., 2007, 2009), or more complex visual tasks involving for example perceptual learning, the establishment of visual routines, or the recognition of complex objects (e.g., Rao and Ballard, 1999; Riesenhuber and Poggio, 1999; Roelfsema et al., 2003), but what is missing in these interesting approaches is a detailed integration of the purely visual subtasks into a complete cognitive behavior.

This situation is reminiscent of the development of the field of artificial intelligence during the last 40 years. After very broad and general claims and initial successes in solving various particular problems (e.g., chess playing, theorem proving) in isolation by particular methods, the community started to ask for more integrated solutions demonstrating the embedding of “symbolic” artificial intelligence-methods into a broader behavioral context [called *symbol grounding* (Harnad, 1990) or *embodiment*], the generalization of solutions from just one particular and often artificial type of problem to a wide variety of naturally occurring or “real world” problems (Artificial General Intelligence^2^), and the development of so-called *cognitive architectures* (e.g., Anderson, 1983, 2007; Newell, 1990; Laird, 2012) which can be used to realize these methods and solutions using well-established building blocks from cognitive psychology (e.g., Wickelgreen, 1979). Also this development points in the direction of more integrated behavioral approaches and perhaps even the use of neural or brain-like structures and processes in the realization of complex cognitive tasks possibly involving symbolic information processing (*neuro-symbolic* integration^3^).

Of course, the realization of serious cognitive abilities or of artificial intelligence, with brain-like neural networks is a hard task, since it requires an understanding and design of networks at the *system level*, and complete cognitive tasks typically involve a substantial part of the whole brain and in particular of the cerebral cortex (Palm et al., 2014), so that we cannot restrict our modeling to a relatively small subnetwork or subsystem. However, this kind of modeling and understanding is definitely needed even in medicine when we want to model for example the use and effect of drugs in the treatment of central neurological, psychiatric or psychological disorders. We will be able to improve medical treatments substantially when we know in more detail the effects of the application of a drug, neurotransmitter or -modulator, at a particular location in the brain, maybe even at particular neurons or particular (types of) synapses.

On the experimental side, the new field of cognitive neuroscience (e.g., Baars and Gage, 2010), which emerged during the “decade of the brain” (the 1990s), could have complemented this new direction of neural information processing theory, but it rather increased the terminological confusion. Neuroscientists who had previously refrained from addressing concepts like consciousness, began discussing its localization in the brain based on the new technique of fMRI, which led to a revival of brain localization of higher cognitive functions in thousands of experimental studies and of philosophical debates about consciousness (e.g., Koch and Tononi, 2011; Tononi, 2012) and cognition in animals and for example, the attribution of some commonsense psychology to monkeys, using the strange label of “theory of mind” (Call and Tomasello, 2008). Of course, all sorts of animals are able to show very intriguing and sophisticated kinds of behavior, but even if it may be fashionable, it is not generally useful to call it “cognitive”. If we want to study human cognitive abilities like language understanding, we can at best do it in animals that are evolutionary close to us. Neurophysiology in humans is possible by non-invasive methods like EEG and fMRI, but fMRI does not provide the spatial and temporal resolution to study in detail how a computation is performed, it only allows to narrow down where it is performed. Among other things, these experiments do not tend to substantiate localist claims, since it is not at all obvious, where to localize consciousness, working memory, language understanding and most components of cognitive architectures in the brain (e.g., Sarter et al., 1996; Uttal, 2003; Ranganath et al., 2004; Ranganath and Blumenfeld, 2005; Kiefer and Pulvermüller, 2012; Pulvermüller et al., 2014; Ulrich et al., 2014). This does not contradict the possibility of modularity in brain organization (Fodor, 1983), but it still remains unclear, what these modules might be (beyond sensory modalities, for example) and how they relate to the particular modules often postulated in mainstream cognitive psychology.

Based on these developments leading to the present state of affairs, it should now be the time to further the theoretical understanding of complex cognitive abilities, including computationally demanding tasks as in artificial intelligence and psychologically and socially important faculties like introspection, empathy, consciousness and free will. The development of such theories should be guided or constrained by our accumulated knowledge from neuroscience, psychology, and computer science.

In order to foster the advancement of computational neuroscience in this direction, it may be useful, but it is certainly not sufficient to organize the collection and distribution of more complete and better experimental neuroscientific data in order to model these data (as in the HBP^1^ or the BAM project, Alivisatos et al., 2012), because this will at best lead to a biophysical understanding of brain activity. In addition, it is necessary to develop synthetic ideas of how certain cognitive abilities involving image or language understanding, planning and non-factual reasoning could be realized adequately in brain-like neural networks, i.e., to understand the neural mechanisms for deliberate decision making and the sequential concerted organization of massively parallel computations (Palm and Bonhoeffer, 1984), or to develop artificial intelligence in brain-like neural networks (see Palm, 1982 for an early attempt).

Kahnemann (2011) has distinguished two kinds of processes that are involved in decision making: *slow* and *fast*. Many complex behavioral abilities, for example in perception, reinforcement learning and motor control, have been understood quite well in computational neuroscience so far, but they typically deal with the “*fast system*” that we share with many animals. The “*slow system*” of decision making which is related to “mental energy” (which requires physical energy, but may not be exactly the energy consumption measured in functional MRI) and interacts in interesting and subtle ways with the fast system, is not easily amenable to neuroscience and has hardly been studied or modelled in computational neuroscience, although we cannot deny its psychological reality. It would be a good candidate for the “conductor” mentioned in the title. Psychological models of these processes (e.g., Anderson, 1983, 2005; Baddeley et al., 1996; Baddeley, 2007; Lewandowsky and Farrell, 2010; Cooper, 2013) are able to describe some of this, but are still far from detailed neurocomputational realizations (Barak and Tsodyks, 2014). If we take cognition seriously and not just use it as a fancy label, we will open a new emerging field of interdisciplinary research between computer science, neuroscience and cognitive psychology.

Criteria for a good neurocomputational cognitive model can be combined from criteria already demanded by neuroscientists, computer scientists and psychologists; some of them that immediately come to mind, are listed below. Certainly any good cognitive model should address several of these criteria.

The basic demand is of course that the model really solves a cognitive task. For this we need a behavioral description of the task, an outline of the solution and a computer program or simulation of it that can be tested on a variety of problem instances. This program should be realized in (or demonstrably convertible into) a neural network architecture. Based on this we can produce a list of criteria:

1. Scalability
2. Efficiency (in real time with realistic size)
3. Neural plausibility
4. Introspective plausibility
5. Reusability (the model should be usable for several related problems)
6. Evolutionary plausibility (how could it have evolved?)
7. Learnability (how could it be learned?)
8. Degradability (it should not immediately break down — “graceful degradation”).

Perhaps in this new kind of large-scale or system-level computational modeling some of the recent developments in the application oriented branch of neural information processing need to be reunited with the neuroscience oriented branch. After all, during evolution the development of intriguing cognitive abilities in the human brain has been pushed forward by the need to solve various complex tasks in the real world by reorganizing the same basic neural machinery. So in order to understand the concerted cooperation of several cortical areas and subcortical structures in the solution of complex cognitive tasks it may in fact be useful to consider the more sophisticated network architectures and learning schemes that have recently been put forward in order to solve complex practical problems in various fields of applications.

## Conflict of Interest Statement

The author declares that the research was conducted in the absence of any commercial or financial relationships that could be construed as a potential conflict of interest.

## Footnotes

^1^humanbrainproject.eu, artificialbrains.com

^2^agi-society.org

^3^neural-symbolic.org
